# Augmentation of trauma-focused psychotherapy for post-traumatic stress disorder: a protocol for a systematic review and meta-analysis

**DOI:** 10.1136/bmjopen-2024-090571

**Published:** 2025-05-21

**Authors:** Lisa Mewes, Till Langhammer, Jonathan Torbecke, Johannes Caspar Fendel, Ulrike Lueken

**Affiliations:** 1 Department of Psychology, Humboldt-Universitat zu Berlin, Berlin, Germany; 2 PartnerSite Berlin/Potsdam, Deutsches Zentrum für Psychische Gesundheit, Berlin, Germany; 3 Department of Psychosomatic Medicine and Psychotherapy, Medical Faculty, Medical Center, University of Freiburg, Freiburg im Breisgau, Germany

**Keywords:** Systematic Review, Meta-Analysis, MENTAL HEALTH, PSYCHIATRY, TRAUMA MANAGEMENT

## Abstract

**Introduction:**

Despite the established status of trauma-focused psychotherapy (TFP) as a first-line treatment for post-traumatic stress disorder (PTSD), a substantial proportion of individuals do not achieve clinically significant improvement or discontinue treatment. Exploring augmentation strategies to enhance treatment outcomes is essential to reduce the overall burden PTSD puts on individuals and society. This protocol outlines a systematic review and meta-analysis of randomised controlled trials (RCTs) evaluating the efficacy of non-pharmacological augmentation strategies in addition to TFP for PTSD treatment.

**Methods and Analysis:**

We comprehensively searched PubMed, Embase, CENTRAL, PTSDpubs, PsycArticles, PsycINFO, PSYNDEX and CINAHL for RCTs without restrictions on publication dates or languages in October 2024. Study screening is currently ongoing. Additionally, we will perform forward and backward searches of the included studies and relevant reviews. Two reviewers will independently screen and select studies, extract data and assess the risk of bias. We will conduct a narrative review to qualitatively synthesise data and a meta-analysis to quantitatively compare the treatment efficacy of augmented TFP with TFP alone or TFP plus placebo. Primary outcomes will be both symptom severity and response rates. The secondary outcome will be dropout rates. We will explore sources of between-study heterogeneity and potential moderators through subgroup and meta-regression analyses. We will assess the overall quality of the included studies with the Grading of Recommendations Assessment, Development, and Evaluation system.

**Ethics and dissemination:**

Ethical approval is not required. We intend to publish results in a peer-reviewed journal and provide materials and data through the Open Science Framework.

**PROSPERO registration number:**

CRD42024549435.

**STRENGTHS AND LIMITATIONS OF THIS STUDY**

- We employ a comprehensive search strategy across eight bibliographic databases that include grey literature to maximise sensitivity in study identification.
- We will evaluate three key outcomes – symptom severity, response rates and dropout rates – while exploring potential moderators through subgroup analyses and meta-regression.
- We will systematically assess individual study quality and overall strength of evidence through standardised evaluation tools, including the second version of the Cochrane Risk of Bias tool and the Grading of Recommendations Assessment, Development and Evaluation system.
- We will include only randomised controlled trials to strengthen internal validity, mitigate bias and enhance the reliability of our findings.
- However, this approach excludes valuable insights from alternative study designs and could limit generalisability of results to real-world clinical settings.

## Introduction

### Rationale

 Post-traumatic stress disorder (PTSD) is a prevalent and serious condition resulting from traumatic experiences, leading to health risks and societal costs that necessitate effective treatment. Most individuals experience at least one traumatic event during their lifetime, with varying exposure rates across nations and specific demographics, such as military personnel.^1^ The risk of developing PTSD following a traumatic event, defined as ‘actual or threatened death, serious injury, or sexual violence’,^2^ is estimated at approximately 4%.^3^ The risk varies depending on the type of trauma experienced, with risk rates up to 19% following interpersonal trauma incidents such as rape.^4^ Beyond the substantial burden of PTSD symptoms, the disorder is associated with numerous adverse outcomes, including increased mortality rate,^5^ elevated risks of cardiovascular and metabolic diseases^6^ as well as substantial societal costs, for example, due to healthcare and unemployment.^7^ Untreated PTSD often persists for many years,^8^ underscoring the need for effective treatments to address the individual and societal burden associated with the disorder.

Available first-line treatments are effective for many PTSD patients, but their impact remains insufficient. International treatment guidelines uniformly recommend trauma-focused psychotherapy (TFP) as the first-line treatment for PTSD.^9 10^ The umbrella term ‘TFP’ encompasses various trauma-focused evidence-based treatments such as variants of trauma-focused cognitive behavioural therapy (TF-CBT) and eye movement desensitisation and reprocessing (EMDR).^10 11^ While their specific treatment rationales may differ, TFPs typically target trauma memories, trauma-related thoughts and emotions and responses to environmental triggers using exposure-based and/or cognitive techniques. These interventions share several core therapeutic mechanisms: fear extinction learning, cognitive processing and restructuring and emotional processing of the traumatic experience.^12^ Several meta-analyses^13^,^16^ report moderate-to-large effect sizes for TFPs in reducing PTSD symptom severity compared with different control groups, including active psychological therapies (eg, supportive psychotherapy), treatment-as-usual and waitlist controls. These treatment effects demonstrate stability over time, indicating enduring efficacy.^17 18^ Conversely, recommendations for pharmacotherapy vary,^9 10^ reflecting smaller sustained treatment effects for pharmacotherapy compared with TFP.^13 19^ However, dropout in TFPs remains a concern, with meta-analyses reporting that approximately one-fifth of patients discontinue TFP treatment.^20 21^ Findings on dropout patterns vary: while Lewis et al.^14^ found higher dropout rates for TFP (18%) than for non-TFP (14%), Varker et al.^20^ reported similar dropout rates between TFPs and active control conditions overall (20.9% vs 20.3%), though rates were notably higher for TFPs in military populations (32.9% vs 23.3%). Further, inadequate treatment response remains a significant challenge at the individual patient level.^22 23^ The definition of treatment response varies across PTSD treatment trials but is most commonly operationalised as loss of diagnosis or reduction of PTSD symptom severity.^24^ Meta-analyses report different response rates for TFP ranging from 35% using standardised 50% symptom reduction thresholds^23^ to 59% when aggregating studies’ diverse author-defined criteria.^22^ Recent systematic evidence suggests that treatment response to TFP is moderated by diverse factors, including biological mechanisms, comorbidities, cognitive functioning, social support and trauma characteristics.^25^ Although these findings primarily reflect correlational relationships with mean post-treatment symptom severity, they suggest that a ‘one-size-fits-all’ approach to PTSD treatment is insufficient. Enhancement of standard TFP approaches appears necessary for the substantial proportion of patients who currently derive insufficient benefit.

Current research addresses inadequate treatment response to TFP by developing and evaluating additional treatment components, often referred to as augmentation strategies. The recently introduced four-stage model for PTSD chronification and treatment^26^ proposes matching interventions to the progression of the disorder – from early neuroprotective strategies in subsyndromal stages to complex, multi-modal treatments for chronic presentations. This model suggests integrating augmentation strategies to enhance established first-line treatments, particularly for patients at risk of non-response, who may be identified early in TFP treatment through promising personalised care approaches.^27 28^ Recent systematic reviews and meta-analyses have indicated that most previously examined pharmaco-agents (eg, selective serotonin reuptake inhibitors and D-cycloserine) have yet failed to demonstrate a robust augmentation effect.^29^,^31^ These limited effects may reflect that pharmacological approaches often target specific fear extinction mechanisms,^31^ potentially overlooking the complex nature of treatment response.^25^ In contrast, non-pharmacological augmentation strategies could offer advantages through their more comprehensive therapeutic pathways: First, they could enhance different core TFP mechanisms, as demonstrated, for example, by exercise promoting neural plasticity^32^ and breathing feedback supporting emotional arousal regulation during exposure.^33^ Second, they could address additional therapeutic targets not explicitly included in TFPs and hereby interact with the overall therapy process rather than specific mechanisms, such as sleep-directed interventions addressing common comorbid difficulties.^34^ To the best of our knowledge, two systematic reviews^31 35^ compiled evidence on non-pharmacological augmentation strategies in TFP. However, at the time of their investigations, most of these strategies had been evaluated in a single randomised controlled trial (RCT) only, with no accompanying meta-analysis conducted. Given the increasing number of RCTs examining integrative, complementary and alternative treatments for PTSD,^36^ updating the existing reviews and evaluating proposed categories of augmentation strategies is warranted. To examine whether non-pharmacological augmentation strategies can improve the treatment outcomes for patients who typically do not respond to TFP or drop out of treatment, it is essential to assess not only the mean symptom reduction but also response rates and dropout rates. However, these effects are unlikely to be uniform across all conditions, as the success of augmentation strategies may depend on factors such as type, dosage, integration within TFP and patient characteristics. To gain insights into for whom and under which conditions what specific augmentation could be most effective, we will explore potential moderators.

### Objectives

Through a systematic review and meta-analysis, we aim to fill this gap by offering researchers, practitioners and policymakers a comprehensive overview of non-pharmacological augmentation strategies in addition to TFP for PTSD. We will explore the characteristics of different non-pharmacological augmentation strategies evaluated in RCTs and assess their overall effect on different aspects of the treatment efficacy for PTSD. Specifically, we will evaluate whether augmentation strategies lead to reduced post-treatment PTSD symptom severity, increased response rates and reduced dropout rates. We will carefully analyse potential moderators and factors contributing to between-study heterogeneity, including different types of augmentation strategies, TFPs, trauma types, treatment resistance at baseline, treatment delivery format, treatment setting, control type, age group, length and dosage of both treatment components (TFP and augmentation).

## Methods

This protocol adheres to the guidelines for Reporting Items for Systematic Review and Meta-Analysis Protocols (PRISMA-P)^37^, and the PRISMA-P checklist^38^ is provided in the online supplemental digital appendix 1. The subsequent systematic review and meta-analysis will comply with the updated PRISMA guidelines.^39^ The study is registered with the International Prospective Register of Systematic Reviews (PROSPERO registration number CRD42024549435). Important amendments to this protocol will be documented within PROSPERO.

### Eligibility criteria

The population, intervention, comparison, outcome and study design framework was used to determine the inclusion and exclusion criteria, as outlined in table 1. TFPs eligible for this review will include established treatments endorsed by current international PTSD treatment guidelines^9 10^: TF-CBTs, EMDR, cognitive processing therapy, prolonged exposure and other exposure-driven approaches specifically addressing trauma memories or environmental triggers (eg, narrative exposure therapy). In this review, augmentation is defined as any (non-pharmacological) intervention ‘delivered prior to, concurrently, or after a first-line PTSD treatment, where the focus of the augmentation was to improve PTSD symptoms and/or improve readiness for treatment, engagement or retention in the first-line treatment’.^31^ The primary outcomes comprise (1) post-intervention PTSD symptom severity compared between groups (augmented TFP vs TFP only/placebo) and (2) response rates based on individual pre-to-post treatment changes. Follow-up data will be examined in sensitivity analyses to assess the durability of treatment effects. If studies used multiple measures to assess symptom severity, the studies’ primary outcome will be used. We anticipate that the included RCTs of first-line PTSD treatments will have varying definitions of treatment response.^24^ Following Cuijpers et al,^23^ we will estimate the number of responders from the means and SD using the method by Furukawa et al.^40^ Following Varker et al,^24^ who recommended thresholds between 30% and 50% symptom reduction, response is defined as a 30% reduction in baseline symptom severity to ensure comparability across different measures while maintaining sensitivity. Sensitivity analyses will be conducted using the response rates as defined in the respective study and using a more conservative 50% symptom reduction threshold, aligned with recent meta-analytical approaches.^23^ As the secondary outcome, dropout rates will be assessed. Following recent meta-analyses,^20 21^ the number of dropouts will be calculated as the difference between the number of randomised participants and those providing post-treatment assessment data.

**Table 1:** Inclusion and exclusion criteria

Criterion	Inclusion criteria	Exclusion criteria
Population	Patients with PTSD diagnosed by a clinician according to DSM or ICD criteria; no or stable concurrent psychotropic medication	Non-clinical or undiagnosed samples; samples in which PTSD is not the primary diagnosis; changes in psychotropic medication during the trial
Intervention	TFP combined with at least one additional non-pharmacological treatment component (augmentation)	No TFP; pharmacological augmentation strategy only
Comparator	TFP only or with a placebo-augmentation control condition	No TFP; augmentation strategy only
Outcomes	PTSD symptom severity, assessed with a clinician-administered PTSD scale (eg, CAPS-5^52^) or validated self-reports (eg, PCL-5^53^)	Self-reports without validation
Study design	Randomised controlled trials	Non-randomised trials, including non-controlled before–after studies, case–control studies, single/clinical case studies, systematic reviews and meta-analyses

CAPS, clinician-administered PTSD scale; DSM, diagnostic and statistical manual of mental disorders; ICD, international classification of disease; PCL, PTSD checklist; PTSD, post-traumatic stress disorder; TFP, trauma-focused psychotherapy.

### Search strategy

We systematically searched the following electronic bibliographic databases: PubMed, Embase (Ovid Interface), Cochrane Register of Controlled Trials (CENTRAL), PTSDpubs (ProQuest interface), PsycArticles, PsycINFO, PSYNDEX and CINAHL (the latter four via the EBSCOhost interface), without restrictions regarding publication date or language in October 2024. The completion of the systematic review is anticipated for the second half of 2025. Given the absence of standardised terminology in the emerging field of augmentation approaches in addition to psychotherapy treatments (eg, augmentation, enhancement and add-on), high sensitivity will be prioritised in our search strategy. Our search terms will be related to (1) PTSD, (2) evidence-based TFPs and (3) RCT study design. Search syntaxes for all bibliographic databases are provided in the online supplemental digital appendix 2. Additionally, we will conduct backward-and-forward literature searches of the included studies and relevant reviews on PTSD treatment. If full texts are unavailable or pertinent information within the scope of this systematic review and meta-analysis is missing, we will contact the corresponding authors of the respective studies and wait 8 weeks for their response. Further, we will reach out to all corresponding authors of the included studies to identify any existing but unpublished data. This applies to both published studies with missing data and to identified study protocols, conference abstracts or trial registrations, for which we will contact investigators to inquire about study completion and data availability.

### Study selection

The study selection process began in November 2024 and is currently ongoing. Two reviewers (LM and TL) are independently conducting software-based study selection. If either reviewer considers the study eligible based on title and abstract screening, we proceed with comprehensive full-text screening. Finally, studies are included when a consensus is reached. Otherwise, a third reviewer is consulted (UL). We document the study selection process in a flowchart adhering to the updated PRISMA guidelines.^39^

### Data extraction

One reviewer (LM) will extract relevant information from the eligible studies. A second reviewer (TL) will independently extract data from a random sample of 10 studies to ensure reliability and minimise bias. A standardised extraction form will be used, which will be pilot-tested and revised if necessary. Information to be extracted include the following: (1) study, encompassing authors, publication year, country, TFP type and control type; (2) population: sample size (at randomisation and pre- and post-treatment assessment), age, sex, ethnicity, trauma type, comorbidities, medication, treatment resistance at baseline (study-defined status, criteria and measures used), inclusion and exclusion criteria; (3) intervention and the comparator: delivery format (face-to-face/online/hybrid), setting (individual/group), augmentation strategy, proposed mechanism of augmentation, treatment characteristics for TFP and augmentation strategy (including session duration, number and frequency of sessions, treatment duration, homework, schedule (prior to, concurrently, or after TFP)), use of measurement tools for intervention integrity including adherence to the protocol and clinical and programme experience of the facilitating therapist; (4) outcomes: means and SD for PTSD symptoms (pre- and post-treatment and follow-up if assessed), time points of assessment, measure of symptom severity, response rates (including absolute numbers of response/non-response), response operationalisation, dropout reasons and adverse events.

### Risk of bias and quality assessment in individual studies

Two reviewers (LM and TL) will independently assess the risk of bias in the included studies using the second version of the Cochrane risk-of-bias assessment tool for randomised trials (RoB2).^41^ Any discrepancies will be resolved through discussion; if no consensus can be reached, a third reviewer will be consulted (UL). Within the RoB2 tool, the RoB is rated as ‘low RoB’, ‘some concerns’, or ‘high RoB’ regarding five domains: (1) bias arising from the randomisation process, (2) deviations from the intended interventions, (3) missing outcome data, (4) measurement of the outcome and (5) selection of the reported result. Based on the rating in these domains, an overall rating is derived for each outcome.

### Risk of bias across studies

To examine publication bias, we will visually inspect funnel plots, compute Egger’s regression test^42^ and Rosenthal’s fail-safe N^43^ and conduct the ‘trim-and-fill’ method.^44^ We will use the Grading of Recommendations Assessment, Development and Evaluation (GRADE) approach^45^ to assess the overall quality of evidence for each outcome, separately for different augmentation approaches and across all included studies. Two reviewers (LM and TL) will independently rank the quality of evidence as ‘high’, ‘moderate’, ‘low’, or ‘very low’ for each of the following dimensions: (1) RoB, (2) inconsistency of results, (3) indirectness of evidence, (4) imprecision of effect size and (5) publication bias.

### Data synthesis

A narrative review will be performed to qualitatively synthesise data on the key characteristics of the included studies. This synthesis will involve categorising augmentation strategies into relevant groups based on proposed frameworks^28^ and insights from the reviewed literature. Building on Metcalf *et al*,^31^ we aim to categorise augmentation strategies based on their proposed primary mechanisms of action. If mechanism-based categorisation proves challenging, alternative organising principles, such as application methods, will be considered to ensure meaningful clinical distinctions. Pertinent results will be reported in a comprehensive ‘summary of findings’ table.

Quantitative data synthesis will be conducted to generate pooled effect sizes for augmentation effects and examine between-study heterogeneity. Owing to the inclusion of studies with diverse characteristics, considerable between-study heterogeneity is anticipated. This assumption will be evaluated by calculating the Q-test, I^2^-statistic^46^ and prediction intervals^47^ within a random-effects meta-analytical framework. To quantify the standardised mean difference for symptom severity between groups (augmented TFP vs TFP only/with placebo) at post-treatment, we will calculate pooled Hedges’ *g*.^48^ For between-group differences in response and dropout rates, we will calculate pooled risk ratios. All effect sizes will be calculated with their 95% CI and associated *p* values. Sensitivity analyses will address RoB, follow-up outcomes, study sample (intention-to-treat vs completers), different response operationalisations and outliers using the ‘non-overlapping CI’ approach.^49^ We will explore sources of heterogeneity with subgroup and meta-regression analyses using mixed-effects models, if we can include at least 10 studies in total and three studies per subgroup.^50^ Subgroups will be formed regarding the augmentation approach, TFP approach, trauma type, treatment resistance at baseline, treatment delivery format, treatment setting, control type and age group. Potential continuous moderators, such as the length and dosage of both treatment components (TFP and augmentation), will be examined using meta-regression analyses. Analyses with insufficient available studies (defined as fewer than 10 times the number of planned analyses) will be considered as exploratory and used to generate hypotheses for future research rather than to draw definitive conclusions. All analyses will be conducted in RStudio.^51^

### Patient and public involvement

Neither patients nor the public will be involved in the study design, conduct, reporting or dissemination plans of this research.

## Ethics and dissemination

Ethical approval is not considered necessary for this study. Results will be published in peer-reviewed journals. We will provide materials and data within the Open Science Framework.

## Supplementary material

10.1136/bmjopen-2024-090571 online supplemental file 1

10.1136/bmjopen-2024-090571 online supplemental file 2
