# Supplementary material for: Augmentation of trauma-focused psychotherapy for post-traumatic stress disorder: a protocol for a systematic review and meta-analysis
Source: BMJ Open. 2025 May 21;15(5):e090571. doi: 10.1136/bmjopen-2024-090571 (PMC12096967; doi:10.1136/bmjopen-2024-090571)
Supplement: online supplemental file 2 [file bmjopen-15-5-s002.pdf]

Supplemental Digital Appendix 2

Search Strategy for PubMed, Embase, CENTRAL, PTSDpubs, PsycArticles, PsycINFO, PSYNDEX, and CINAHL

PubMed

No.	Search Terms
#1	PTSD [tiab] OR “posttraumatic stress disorder” [tiab] OR “post traumatic stress disorder” [tiab] OR stress disorders, post traumatic [mesh]
#2	“trauma focused” [tiab] OR psychotherap* [tiab] OR “cognitive therapy” [tiab] OR “cognitive behavio* therapy” [tiab] OR CBT [tiab] OR “cognitive processing therapy” [tiab] OR CPT [tiab] OR “exposure therapy” [tiab] OR “prolonged exposure” [tiab] OR “narrative exposure” [tiab] OR “eye movement desensiti*” [tiab] OR EMDR [tiab] OR Psychotherapy [mesh]
#3	RCT [tiab] OR”clinical trial” [tiab] OR “parallel design” [tiab] OR “controlled trial” [tiab] OR randomi* [tiab] OR randomly [tiab] OR “treatment trial” [tiab] OR Randomized Controlled Trials [Publication Type]
#4	#1 AND #2 AND #3

Note. We include textwords in title and abstract (tiab) and keywords with controlled vocabulary (MeSH terms).

Embase via Ovid

No.	Search Terms
#1	(PTSD or "posttraumatic stress disorder" or "post traumatic stress disorder").ti,ab. or exp posttraumatic stress disorder/
#2	("trauma focused*" or psychotherap* or "cognitive therapy" or "cognitive behavioural therapy" or CBT or "cognitive processing therapy" or CPT or "exposure therapy" or "prolonged exposure" or "narrative exposure" or "eye movement desensiti*" or EMDR).ti,ab. OR exp trauma-focused cognitive behavioral therapy/ OR exp cognitive processing therapy/ OR exp exposure therapy
#3	(RCT or "clinical trial" or "parallel design" or "controlled trial" or randomi* or randomly or "treatment trial").ti,ab. or exp randomized controlled trial/
#4	#1 AND #2 AND #3

Note. We include text words in title and abstract (.ti,ab.) and keywords with controlled vocabulary (Emtree terms).

CENTRAL

No.	Search Terms
#1	PTSD OR “posttraumatic stress disorder” OR “post traumatic stress disorder”
#2	"trauma focused" OR psychotherap* OR "cognitive therapy" OR cognitive NEXT behavio* NEXT therapy OR CBT OR "cognitive processing therapy" OR CPT OR "exposure therapy" OR "prolonged exposure" OR "narrative exposure" OR eye NEXT movement NEXT desensiti* OR EMDR
#3	RCT OR “clinical trial” OR “parallel design” OR “controlled trial” OR randomi* OR randomly OR “treatment trial”
#4	#1 AND #2 AND #3

Note. We include text words in title, abstract, and keywords.

PTSDpubs via ProQuest

No.	Search Terms
#1	PTSD or "posttraumatic stress disorder" or "post traumatic stress disorder"
#2	"trauma focused*" or psychotherap* or "cognitive therapy" or "cognitive behavio* therapy" or CBT or "cognitive processing therapy" or CPT or "exposure therapy" or "prolonged exposure" or "narrative exposure" or "eye movement desensiti*" or EMDR
#3	RCT or "clinical trial" or "parallel design" or "controlled trial" or randomi* or randomly or "treatment trial"
#4	#1 AND #2 AND #3

Note. We include text word search in all fields except from full text (noft).

PsycArticles, PsycInfo, PSYNDEX via EBSCOhost

No.	Search Terms
#1	TI "ptsd" OR AB "ptsd" OR TI "posttraumatic stress disorder" OR AB "posttraumatic stress disorder" OR TI "post traumatic stress disorder" OR AB "post traumatic stress disorder" OR DE "Posttraumatic Stress Disorder" OR DE "Complex PTSD"
#2	TI "trauma focused" OR AB "trauma focused" OR TI "psychotherap*" OR AB "psychotherap*" OR TI "cognitive therapy" OR AB "cognitive therapy" OR TI "cognitive behavio* therapy" OR AB "cognitive behavio* therapy" OR TI “cbt” or AB “cbt” OR TI "cognitive processing therapy" OR AB "cognitive processing therapy" OR TI “cpt” OR AB “cpt” OR TI "exposure therapy" OR AB "exposure therapy" OR TI "prolonged exposure" OR AB "prolonged exposure" OR TI "narrative exposure" OR AB "narrative exposure" OR TI "eye movement desensiti*" OR AB "eye movement desensiti*" OR TI "EMDR" OR AB "EMDR" OR - DE "Psychotherapy" OR DE "Trauma Treatment" OR DE "Trauma-Focused Cognitive Behavior Therapy" OR DE "Cognitive Behavior Therapy" OR DE "Cognitive Therapy" OR DE "Behavior Therapy" OR DE "Exposure Therapy" OR DE "Imaginal Exposure" OR DE "Prolonged Exposure Therapy" OR "Narrative Therapy" OR DE "Eye Movement Desensitization Therapy"
#3	TI "rct" OR AB "rct" OR TI "clinical trial" OR AB "clinical trial" OR TI "parallel design" OR AB "parallel design" OR TI "controlled trial" OR AB "controlled trial" OR TI "randomi*" OR AB "randomi*" OR TI "randomly" OR AB "randomly" OR TI "treatment trial" OR AB "treatment trial" OR DE "Randomized Controlled Trials" OR DE "Randomized Clinical Trials"
#4	#1 AND #2 AND #3

Notes. We include text words in title (TI) and abstract (AB) and keywords with controlled vocabulary (DE).

CINAHL via EBSCOhost

No.	Search Terms
#1	TI "ptsd" OR AB "ptsd" OR TI "posttraumatic stress disorder" OR AB "posttraumatic stress disorder" OR TI "post traumatic stress disorder" OR AB "post traumatic stress disorder"OR MH "Stress Disorders, Post-Traumatic+"
#2	TI "trauma focused" OR AB "trauma focused" OR TI "psychotherap*" OR AB "psychotherap*" OR TI "cognitive therapy" OR AB "cognitive therapy" OR TI "cognitive behavio* therapy" OR AB "cognitive behavio* therapy" OR TI “cbt” or AB “cbt” OR TI "cognitive processing therapy" OR AB "cognitive processing therapy" OR TI “cpt” OR AB “cpt” OR TI "exposure therapy" OR AB "exposure therapy" OR TI "prolonged exposure" OR AB "prolonged exposure" OR TI "narrative exposure" OR AB "narrative exposure" OR TI "eye movement desensiti*" OR AB "eye movement desensiti*" OR TI "EMDR" OR AB "EMDR" OR MH "Psychotherapy+"
#3	TI "rct" OR AB "rct" OR TI "clinical trial" OR AB "clinical trial" OR TI "parallel design" OR AB "parallel design" OR TI "controlled trial" OR AB "controlled trial" OR TI "randomi*" OR AB "randomi*" OR TI "randomly" OR AB "randomly" OR TI "treatment trial" OR AB "treatment trial" OR MH "Randomized Controlled Trials+"
#4	#1 AND #2 AND #3

Notes. We include text words in title (TI) and abstract (AB) and keywords with controlled vocabulary (MH+).
