# Supplementary material for: Augmentation of trauma-focused psychotherapy for post-traumatic stress disorder: a protocol for a systematic review and meta-analysis
Source: BMJ Open. 2025 May 21;15(5):e090571. doi: 10.1136/bmjopen-2024-090571 (PMC12096967; doi:10.1136/bmjopen-2024-090571)
Supplement: Supplementary file 3 [file bmjopen-2024-090571.reviewer_comments.pdf]

PEER REVIEW HISTORY

BMJ Open publishes all reviews undertaken for accepted manuscripts. Reviewers are asked to complete a checklist review form and are provided with free text boxes to elaborate on their assessment. These free text comments are reproduced below.

ARTICLE DETAILS

Title (Provisional)

Augmentation of trauma-focused psychotherapy for post-traumatic stress disorder: A protocol for a systematic review and meta-analysis.

Authors

Mewes, Lisa; Langhammer, Till; Torbecke, Jonathan; Fendel, Johannes Caspar; Lueken, Ulrike

---

VERSION 1 - REVIEW

**Reviewer**	**1**
**Name**	**Bryant, Richard**
**Affiliation**	**University of New South Wales, Sydney, Australia**
**Date**	**30-Aug-2024**
**COI**	**NA**

This protocol describes a systematic review and meta-analysis of RCTs that attempt to augment trauma-focused psychotherapy for PTSD. My comments are:

1. The authors should pay more attention to description and definition of Trauma-Focused Psychotherapy. A better description of the interventions encompassed in this term (which they describe in the search terms).

2. Given the study will be on augmentation strategies, it is worth describing the mechanism of TF-psychotherapy, and what core mechanisms have been targeted to augment the effects of treatment. At present the protocol paper is very light on the reasoning for the strategies but these are key to interpreting results. In the field of pharmacological augmentation, nearly all trials have modulated extinction processes. Describe the mechanisms for non-pharmacological.

3. Check for typos (see Table 1).

4. The authors' approach of having at least 10 studies and 3 per subgroup is sound for the purpose of meta-analysis, and consistent with previous meta-analyses.

5. One issue that requires clarification is the inclusion of treatment drop out. This can be problematic because early drop out can occur because of early treatment response and

therefore not indicative of bad outcome. Also, how do they operationalize this construct because studies define drop out variably.

**Reviewer**	**2**
**Name**	**Keyan, Dharani**
**Affiliation**	**UNSW, School of Psychology**
**Date**	**03-Sep-2024**
**COI**	**none**

This manuscript presents a protocol for a systematic review and meta-analysis on augmentation strategies used in the treatment of trauma-focused psychotherapy for PTSD. The purpose of this review is timely within the context of recent empirical studies and recent reviews highlighting the role of varied number of factors that predict PTSD response and ways in which researchers have attempted to target such factors during treatment.

Overall, the paper is well constructed. However, I do suggest that authors proof-read the manuscript for spelling and grammatical errors.

The introduction endeavours to be succinct in its background. It would benefit from more thoughtful reasoning to build a strong rationale for this review. For example, whilst the authors highlight that inadequate treatment response persists as a prevalent issue, they do not provide a reference for this statement. A recent review by Keyan et al. 2024 (https://doi.org/10.1037/bul0000438) systematically highlighted a varied number and type of factors that contribute to varied treatment response (i.e., increased or decreased PTSD severity) following trauma-focused psychotherapy. Please consider citing this or equivalent. The statement in lines 95-97 is unclear as it appears to suggest that inadequate treatment response is defined as a loss of diagnosis or reduction in PTSD symptom severity. Please clarify. In line 104, the authors refer to 'stage models' of PTSD. It is unclear what is meant by this statement.

The methods section requires further attention. Can the authors please provide the PROSPERO registration identification number such that a reader can review the apirori aims and other details pertaining to this review. In lines 147-148 the authors highlight a few eligible forms of trauma-focused psychotherapy. What is unclear is what type of treatment would be considered 'trauma-focused' and what would not. I suggest the authors to define this here such that the inclusion and exclusion criteria pertaining to types of treatment trials is more apparent to the reader. Additionally, in lines 152-153, the authors state that the primary outcome will be post-intervention PTSD severity and response rates; is this a single time-point measurement only? Or will you include change in PTSD severity pre-post/mid-point-post, or even follow-up PTSD response. Please clarify this. For your secondary outcomes, how will you be defining drop-out? What framework or definition will you be using, or will this be data-driven, and if so, how? Section 2.3 has missing details on who will be carrying out the screening and data extraction. If the authors are aware of whom in their team will be conducting this, please provide details. If not, please remove the 'XX and XX' where relevant in this section and throughout the paper (e.g., risk of bias assessments section). In section 2.4,

you state that you will be extracting information relating to treatment resistance at baseline. Can the authors clarify how this will be determined. In section 2.7, I encourage the authors to detail how you will be categorising augmentation strategies into meta-analytic groups that do not fit previous categorisation standards that you have cited in line 230. What framework, or method(s) will be you relying on to guide this categorisation? Given this is an updated review, it will not be surprising if you find augmentation strategies that do not neatly fit prior categorisation standards. Additionally, briefly state how you will be dealing with missing data in the event data is not readily available or cannot be extracted (e.g., primary papers do not provide necessary details for extraction).

In section 2.8 or elsewhere, please state if you will be publishing your data in OSF or equivalent.

However, this is review is a very useful and time contribution to the literature on avenues for augmentation of trauma-focused psychotherapy for PTSD.

**Reviewer**	**3**
**Name**	**Rossouw, Jaco**
**Affiliation**	**Centre for CBT**
**Date**	**08-Feb-2025**
**COI**	**I have no competing interests to declare**

Thank you for the opportunity to review this protocol.

line 88: this is correct, however the triggers in the environment (in-vivo) is also addressed in most TFPs. I believe this is worth mentioning

line 95: the dropout rate of 20% is rather the dropout rate for therapy for PTSD in general and not TFPs specifically, as the dropout rate for active control conditions have been comparable with TFPs in multiple studies. I think the statement in the protocol should indicate this, at this point this stands in isolation as a function of TFPs.

line 98: Given that the authors choose the response rate for their study as a 30% change, elaborating on the findings from studies more and maybe using this as a motivation for their own decision of 30% change in symptoms as a response to treatment.

line 103: I think a more comprehensive discussion on augmentation strategies and how they have been used in the past is in order at this point. The mention of the 2 reviews seems to introductory, given this is the area of focus of this review. Expanding on the role of augmentation and maybe examples of augmentation from existing studies seems appropriate. Also discussing the moderators identified prior to setting the objectives describing the moderators seems indicated.

The methods section is well-written.

I look forward to the results of this review.

VERSION 1 - AUTHOR RESPONSE

Reviewer: 1

Dr. Richard Bryant, University of New South Wales, Sydney, Australia
Comments to the Author:

This protocol describes a systematic review and meta-analysis of RCTs that attempt to augment trauma-focused psychotherapy for PTSD. My comments are:

Reply: We greatly appreciate the thoughtful and constructive feedback provided by Reviewer 1. Their expertise in PTSD research has helped us significantly improve the theoretical foundation and methodological clarity of our protocol.

1. The authors should pay more attention to description and definition of Trauma-Focused Psychotherapy. A better description of the interventions encompassed in this term (which they describe in the search terms).

Reply: Thank you for highlighting the need for a more comprehensive description of trauma-focused psychotherapy (TFP). We have enhanced the definition in the introduction section to provide clearer conceptual background:

„The umbrella term TFP encompasses various trauma-focused evidence-based treatments such as variants of trauma-focused cognitive behavioral therapy (TF-CBT) and eye movement desensitization and reprocessing (EMDR) (Burback et al., 2024; Martin et al., 2021)*. While their specific treatment rationales may differ, TFPs typically target trauma memories, trauma-related thoughts and emotions, and responses to environmental triggers using exposure-based and/or cognitive techniques. These interventions share several core therapeutic mechanisms: fear extinction learning, cognitive processing and restructuring, and emotional processing of the traumatic experience (Schnyder et al., 2015).“*

Additionally, we have added a more detailed operational definition in the methods section (2.1) that directly informs our study selection criteria:

“Eligible TFPs for this review will include established treatments endorsed by current international PTSD treatment guidelines (Hamblen et al., 2019; Martin et al., 2021)*: TF-CBTs, EMDR, cognitive processing therapy (CPT), prolonged exposure (PE), and other exposure-driven approaches specifically addressing trauma memories or environmental triggers (e.g. narrative exposure therapy (NET)).“*

While we maintain a broader overview of TFP in the introduction to establish context, this specific definition in the methods ensures clear and transparent inclusion criteria for the review process.

2. Given the study will be on augmentation strategies, it is worth describing the mechanism of TF-psychotherapy, and what core mechanisms have been targeted to augment the effects of treatment. At present the protocol paper is very light on the reasoning for the strategies but these are key to interpreting

results. In the field of pharmacological augmentation, nearly all trials have modulated extinction processes. Describe the mechanisms for non-pharmacological.

Reply: Thank you for this important suggestion. We have enhanced our theoretical foundation in the introduction by adding descriptions of both TFP core mechanisms (as mentioned in our response to your first comment) and mechanisms for nonpharmacological augmentation approaches:

"These limited effects may reflect that pharmacological approaches often target specific fear extinction mechanisms (Metcalf et al., 2020)*, potentially overlooking the complex nature of treatment response* (Keyan et al., 2024)*. In contrast, nonpharmacological augmentation strategies could offer advantages through their more comprehensive therapeutic pathways: First, they could enhance different core TFP mechanisms, as demonstrated for example by exercise promoting neural plasticity* (Powers et al., 2015)*, and breathing feedback supporting emotional arousal regulation during exposure* (Rosaura Polak et al., 2015)*. Second, they could address additional therapeutic targets not explicitly included in TFPs and hereby interact with the overall therapy process rather than specific mechanisms, such as sleep-directed interventions addressing common comorbid difficulties* (Galovski et al., 2016)*."*

3. Check for typos (see Table 1).

Reply: We have conducted a thorough review of the table, standardized the formatting and corrected all identified typographical errors:

Table 1

Inclusion and exclusion criteria

Criterion	Inclusion criteria	Exclusion criteria
Population	Patients with PTSD diagnosed by a clinician according to DSM or ICD criteria; no or stable concurrent psychotropic medication	Non-clinical or undiagnosed samples; samples in which PTSD is not the primary diagnosis; changes in psychotropic medication during the trial
Intervention	TFP combined with at least one additional nonpharmacological treatment component (augmentation)	No TFP; pharmacological augmentation strategy only
Comparator	TFP only or with a placebo-augmentation control condition	No TFP; augmentation strategy only
Outcomes	PTSD symptom severity, assessed with a clinician-administered PTSD scale (e.g. CAPS-5 (Weathers et al., 2018)) or validated self-reports (e.g. PCL-5 (Blevins et al., 2015))	Self-reports without validation

Study design	Randomized controlled trials	Non-randomized trials, including non-controlled before-after studies, case-control studies, single/clinical case studies, systematic reviews, meta-analyses

Note. CAPS = Clinician-Administered PTSD Scale; DSM = Diagnostic and Statistical Manual of Mental Disorders; ICD = International Classification of Disease; PCL = PTSD checklist; PTSD = Post-traumatic stress disorder; TFP = Trauma-focused psychotherapy.

4. The authors' approach of having at least 10 studies and 3 per subgroup is sound for the purpose of meta-analysis, and consistent with previous meta-analyses.

Reply: Thank you for confirming the appropriateness of our methodological approach.

5. One issue that requires clarification is the inclusion of treatment drop out. This can be problematic because early drop out can occur because of early treatment response and therefore not indicative of bad outcome. Also, how do they operationalize this construct because studies define drop out variably.

Reply: Thank you for bringing attention to the important issue of dropout assessment. You raise a critical point that early discontinuation due to treatment success should not be classified as bad outcome. We fully agree with this perspective and have adopted an operational definition that helps address this concern:

"Following recent meta-analyses (Lewis et al., 2020; Varker et al., 2021)*, we will calculate the number of dropouts as the difference between the number of randomized participants and those providing post-treatment assessment data."*

Please note that this approach helps to distinguish between participants who discontinue participation entirely and those who complete post-treatment assessments despite ending active treatment sessions (potentially due to early improvement). Additionally, we will extract all reported reasons for discontinuation to better understand dropout patterns. We have added dropout reasons as a variable for data extraction (line 247-248) to enable more nuanced interpretation of these findings.

We thank Reviewer 1 once again for their valuable insights and careful review, which have helped enhance both the theoretical grounding and methodological rigor of our protocol.

Reviewer: 2

Dr. Dharani Keyan, UNSW
Comments to the Author:

This manuscript presents a protocol for a systematic review and meta-analysis on augmentation strategies used in the treatment of trauma-focused

psychotherapy for PTSD. The purpose of this review is timely within the context of recent empirical studies and recent reviews highlighting the role of varied number of factors that predict PTSD response and ways in which researchers have attempted to target such factors during treatment.

Reply: We sincerely thank Reviewer 2 for their comprehensive evaluation of our manuscript. Their detailed feedback, particularly regarding the theoretical background for treatment response, methodological precision in outcome definitions, and suggestions for strengthening our analytical framework, has substantially improved our protocol.

1. Overall, the paper is well constructed. However, I do suggest that authors proof-read the manuscript for spelling and grammatical errors.

Reply: We have conducted a thorough proof-read of the manuscript for spelling and grammatical errors.

2. The introduction endeavours to be succinct in its background. It would benefit from more thoughtful reasoning to build a strong rationale for this review. For example, whilst the authors highlight that inadequate treatment response persists as a prevalent issue, they do not provide a reference for this statement.

Reply: Thank you for this valuable point about strengthening our rationale with appropriate citations. We have revised the text to more clearly refer to meta-analytical evidence supporting our statement about the prevalence of inadequate treatment response:

- *"Further, inadequate treatment response remains a significant challenge at the individual patient level (Cuijpers et al., 2024; Loerinc et al., 2015)."*
- *"Meta-analyses report different response rates for TFP ranging from 35% using standardized 50% symptom reduction thresholds (Cuijpers et al., 2024) to 59% when aggregating studies' diverse author-defined criteria (Loerinc et al., 2015)."*

3. A recent review by Keyan et al. 2024 (https://doi.org/10.1037/bul0000438) systematically highlighted a varied number and type of factors that contribute to varied treatment response (i.e., increased or decreased PTSD severity) following trauma-focused psychotherapy. Please consider citing this or equivalent.

Reply: Thank you for bringing attention to the relevant field of predictors of treatment response. We appreciate the reference to Keyan et al. (2024), which was published in June 2024 coinciding with our manuscript submission. This valuable systematic review and meta-analysis provides important insights that we were not aware of at the time of our protocol development.

We have incorporated this reference in our manuscript to strengthen the rationale for augmentation studies where we discuss response rates and factors influencing treatment outcomes:

"Recent systematic evidence suggests that treatment response to trauma-focused psychotherapy is moderated by diverse factors including biological mechanisms, comorbidities, cognitive functioning, social support, and trauma characteristics (Keyan et al., 2024)*. Though these findings primarily reflect correlational*

relationships with mean post-treatment symptom severity, they suggest that a 'one-size-fits-all' approach to PTSD treatment is insufficient. Enhancement of standard TFP approaches appears necessary for the substantial proportion of patients who currently derive insufficient benefit.“

4. The statement in lines 95-97 is unclear as it appears to suggest that inadequate treatment response is defined as a loss of diagnosis or reduction in PTSD symptom severity. Please clarify.

Reply: Thank you for highlighting this unclear phrasing. We agree that our original wording could be misleading. We have revised this sentence to clearly distinguish between treatment response and inadequate response:

“The definition of treatment response varies across PTSD treatment trials but is most commonly operationalized as loss of diagnosis or reduction of PTSD symptom severity (Varker et al., 2020).”

5. In line 104, the authors refer to ‘stage models’ of PTSD. It is unclear what is meant by this statement.

Reply: Thank you for highlighting this need for clarification. We have revised the passage to more precisely explain the concept of stage models:

"The recently introduced four-stage model for PTSD chronification and treatment [23] proposes matching interventions to the progression of the disorder - from early neuroprotective strategies in subsyndromal stages to complex, multi-modal treatments for chronic presentations. This model suggests integrating augmentation strategies to enhance established first-line treatments, particularly for patients at risk of nonresponse, who may be identified early in TFP treatment through promising personalized care approaches [24,25]."

6. The methods section requires further attention. Can the authors please provide the PROSPERO registration identification number such that a reader can review the apirori aims and other details pertaining to this review.

Reply: We had initially anonymized this information in our submission, but following the editor's request, we have now included the full PROSPERO registration number (CRD42024549435) in both the abstract and methods section of our manuscript.

7. In lines 147-148 the authors highlight a few eligible forms of trauma-focused psychotherapy. What is unclear is what type of treatment would be considered ‘trauma-focused’ and what would not. I suggest the authors to define this here such that the inclusion and exclusion criteria pertaining to types of treatment trials is more apparent to the reader.

Reply: Thank you for requesting clarification about the classification of trauma-focused treatments. We have addressed this by adding explicit eligibility criteria for TFPs in section 2.1. Our definition encompasses established, trauma-focused treatments that are endorsed by current clinical practice guidelines:

“Eligible TFPs for this review will include established treatments endorsed by current international PTSD treatment guidelines (Hamblen et al., 2019; Martin et al., 2021)*: TF-CBTs, EMDR, cognitive processing therapy (CPT), prolonged exposure (PE), and other exposure-driven approaches specifically addressing trauma memories or environmental triggers (e.g. narrative exposure therapy (NET)).”*

8. Additionally, in lines 152-153, the authors state that the primary outcome will be post-intervention PTSD severity and response rates; is this a single time-point measurement only? Or will you include change in PTSD severity pre-post/mid-point-post, or even follow-up PTSD response. Please clarify this.

Reply: Thank you for your important question regarding outcome assessment timing. We apologize for any lack of clarity in lines 152-153 and appreciate the opportunity to elaborate on our analytical approach:

For symptom severity, we will compare post-treatment scores between groups (augmented TFP vs. TFP only/placebo) as our primary outcome measure. As noted in the Cochrane Handbook, this approach is methodologically sound for meta-analyzing outcome data from RCTs where the randomization process ensures comparable baseline symptom severity across groups, and analyses of post-intervention measurements typically address the same underlying intervention effects (Deeks et al., 2023).

For response rates, we will examine pre-to-post treatment changes, defining response as a 30% reduction from baseline symptom severity (Varker et al., 2020). This approach allows us to capture clinically meaningful individual-level improvement while maintaining standardization across studies.

To ensure comprehensive evaluation, we will:

- Extract data from all available time points (pre-treatment, post-treatment, follow-up)
- Conduct and report sensitivity analyses incorporating follow-up data where available

We have clarified this in section 2.1:

"The primary outcomes comprise (1) post-intervention PTSD symptom severity compared between groups (augmented TFP vs. TFP only/ placebo), and (2) response rates based on individual pre-to-post treatment changes. Follow-up data will be examined in sensitivity analyses to assess the durability of treatment effects."

9. For your secondary outcomes, how will you be defining drop-out? What framework or definition will you be using, or will this be data-driven, and if so, how?

Reply: Thank you for this important question about dropout definition. This concern was also raised by Reviewer 1, emphasizing both the variability in dropout definitions across studies and the complexity of interpreting dropout. We have now implemented a standardized operational definition in section 2.1:

"Following recent meta-analyses (Lewis et al., 2020; Varker et al., 2021)*, we will calculate the number of dropouts as the difference between the number of randomized participants and those providing post-treatment assessment data."*

Additionally, we will extract all reported reasons for discontinuation to better understand dropout patterns.

10. Section 2.3 has missing details on who will be carrying out the screening and data extraction. If the authors are aware of whom in their team will be conducting this, please provide details. If not, please remove the 'XX and XX' where relevant in this section and throughout the paper (e.g., risk of bias assessments section).

Reply: Thank you for highlighting the need for clarity regarding team member responsibilities. Following the editor's request to remove anonymization, we have now specified the team members responsible for each step of the review process (sections 2.3-2.6):

2.3 Study selection

- *"Two reviewers (LM and TL) are independently conducting software-based study selection."*
- *"If no consensus is reached, a third reviewer is consulted (UL)."*

2.4 Data extraction

- *"One reviewer (LM) will extract relevant information from the eligible studies. A second reviewer (TL) will independently extract data from a random sample 10 studies to ensure reliability and minimize bias."*

2.5 Risk of bias and quality assessment in individual studies

- *"Two reviewers (LM and TL) will independently assess the risk of bias in the included studies using the second version of the Cochrane risk-of-bias assessment tool for randomized trials (RoB2)* (Sterne et al., 2019)*. We will resolve any discrepancies through discussion; if no consensus can be reached, a third reviewer will be consulted (UL)."*

2.6 Risk of bias across studies

- *Two reviewers (LM and TL) will independently rank the quality of evidence as 'high', 'moderate', 'low', or 'very low' for each of the following dimensions: (1) risk of bias, (2) inconsistency of results, (3) indirectness of evidence, (4) imprecision of effect size, and (5) publication bias.*

11. In section 2.4, you state that you will be extracting information relating to treatment resistance at baseline. Can the authors clarify how this will be determined.

Reply: Thank you for this important request for clarification regarding treatment resistance assessment. Treatment resistance is a key consideration in our review, as theoretical frameworks suggest augmentation strategies may be particularly relevant for treatment-resistant populations (Burback et al., 2024; Nijdam et al., 2023). To systematically capture this information, we will: (1) extract whether primary study authors explicitly define their sample as treatment resistant, (2) document the specific criteria used to define treatment resistance (e.g., number of failed previous treatment attempts, inadequate response to ongoing TFP), (3) record any baseline measures or assessments used to quantify treatment resistance. We have clarified this in section 2.4 by modifying the text to read:

"[…] treatment resistance at baseline (study-defined status, criteria, and measures used)"

12. In section 2.7, I encourage the authors to detail how you will be categorising augmentation strategies into meta-analytic groups that do not fit previous categorisation standards that you have cited in line 230. What framework, or method(s) will be you relying on to guide this categorisation? Given this is an updated review, it will not be surprising if you find augmentation strategies that do not neatly fit prior categorisation standards.

Reply: Thank you for bringing attention to this core aspect of our review. We will use a data-driven approach, guided by insights from the included studies on addressed mechanisms, content, modality, application methods and intended health implications, to refine the previously proposed framework for characterizing augmentation strategies. Following your comment, we have added the following clarification to section 2.7:

"Building on Metcalf and colleagues (2020), we aim to categorize augmentation strategies based on their proposed primary mechanisms of action. If mechanism-based categorization proves challenging, alternative organizing principles, such as application methods, will be considered to ensure meaningful clinical distinctions."

This approach acknowledges that while Metcalf et al.'s (2020) framework provides a valuable starting point, our exclusive focus on nonpharmacological strategies, and the inclusion of new studies may require adaptations. For example, categories originally developed for pharmacological augmentation (e.g., first-line combinations, pharmaco-polytherapy) will need to be reconceptualized for nonpharmacological approaches. We will form new categories if we find studies that address mechanistical aspects not represented in the former framework. We recognize that some nonpharmacological interventions may operate through multiple mechanisms simultaneously, in which case we aim to identify the primarily relevant mechanism for classification. If possible, we will further differentiate existing categories in the existing framework, such as "global cognitive enhancers" and "specific cognitive enhancers" if we find multiple studies that form a distinctive subgroup, for example through addressing a specific cognitive aspect (e.g. attention bias) or specific application methods (e.g. neurostimulation, computerized cognitive training).

As prespecified, subgroup analyses will only be conducted if there are a minimum of three studies per subgroup and findings will be framed as preliminary insights to guide future research. We will provide detailed reasoning for our final categorization framework in the systematic review report.

13. Additionally, briefly state how you will be dealing with missing data in the event data is not readily available or cannot be extracted (e.g., primary papers do not provide necessary details for extraction).

Reply: Thank you for raising this important point about handling missing data. We have clarified our approach in section 2.2 by expanding the existing statement about contacting authors. The text now reads:

"If full texts are unavailable or pertinent information within the scope of this systematic review and meta-analysis is missing, we will contact the corresponding authors of the respective studies and wait eight weeks for their response. This applies to both published studies with missing data and to identified study protocols, conference abstracts, or trial registrations, for which we will contact investigators to inquire about study completion and data availability."

14. In section 2.8 or elsewhere, please state if you will be publishing your data in OSF or equivalent.

Reply: Thank you for emphasizing the importance of data transparency. We have specified in both the abstract and the 'Ethics and dissemination' section (section 3) that we will provide materials and data through the Open Science Framework (OSF).

However, this is review is a very useful and time contribution to the literature on avenues for augmentation of trauma-focused psychotherapy for PTSD.

Reply: We sincerely thank Reviewer 2 for their thorough and constructive review. Their detailed methodological suggestions and emphasis on clarity have substantially strengthened our protocol. We particularly appreciate their recognition of our review's contribution to the field of PTSD treatment augmentation. Their feedback has helped us improve the theoretical foundation, methodological precision, and overall transparency of our systematic review protocol.

Reviewer: 3
Dr. Jaco Rossouw, Centre for CBT, Stellenbosch University Faculty of Medicine and Health Sciences

Comments to the Author:

Thank you for the opportunity to review this protocol.

Reply: We thank Reviewer 3 for their thoughtful and constructive suggestions for strengthening the theoretical rational for our review and improving methodological precision.

1. line 88: this is correct, however the triggers in the environment (in-vivo) is also addressed in most TFPs. I believe this is worth mentioning

Reply: Thank you for highlighting the role of environmental triggers in trauma-focused psychotherapy. We agree that making this aspect explicit would enhance clarity. We have modified the relevant sentence to read:

"While their specific treatment rationales may differ, TFPs typically target trauma memories, trauma-related thoughts and emotions, and responses to environmental triggers using exposure-based and/or cognitive techniques."

2. line 95: the dropout rate of 20% is rather the dropout rate for therapy for PTSD in general and not TFPs specifically, as the dropout rate for active control conditions have been comparable with TFPs in multiple studies. I think the statement in the protocol should indicate this, at this point this stands in isolation as a function of TFPs.

Reply: Thank you for this important observation about dropout rates in PTSD treatments. We acknowledge that dropout patterns can be complex and there have been somewhat different findings regarding comparable dropout rates between TFP and active control conditions in recent meta-analyses. Given the relevance of this measure, we have revised the manuscript to reflect these nuances while maintaining our focus on TFP-specific dropout rates:

“However, dropout in TFPs remains a concern, with meta analyses reporting that approximately one-fifth of patients discontinue TFP treatment (Lewis et al., 2020; Varker et al., 2021)*. Findings on dropout patterns vary: while Lewis et al. (2020) found higher dropout rates for TFP (18%) compared with non-TFP (14%), Varker et al. (2021) reported similar dropout rates between TFPs and active control conditions overall (20.9% vs. 20.3%), though rates were notably higher for TFPs in military populations (32.9% vs. 23.3%).“*

3. line 98: Given that the authors choose the response rate for their study as a 30% change, elaborating on the findings from studies more and maybe using this as a motivation for their own decision of 30% change in symptoms as a response to treatment.

Reply: Thank you for your valuable suggestion to elaborate on the empirical basis for our response rate threshold. You raise an important point, as response definitions significantly impact reported treatment outcomes. We have added relevant information to the introduction regarding the definition of treatment response and varying meta-analytical findings in relation with different operationalizations:

“The definition of treatment response varies across PTSD treatment trials but is most commonly operationalized as loss of diagnosis or reduction of PTSD symptom severity (Varker et al., 2020)*. Meta-analyses report different response rates for TFP ranging from 35% using standardized 50% symptom reduction thresholds* (Cuijpers et al., 2024) *to 59% when aggregating studies’ diverse author-defined criteria* (Loerinc et al., 2015)*.”*

Moreover, following recommendations from a systematic review, which suggested using thresholds between 30-50% reduction in baseline symptoms (Varker et al., 2020), we chose 30% as our primary definition to ensure maximum sensitivity while maintaining clinical meaningfulness. To address this methodological challenge comprehensively while ensuring standardization across studies, we will use a more conservative 50% threshold in our sensitivity analyses. We have clarified this in the methods section (2.1) as follows:

“Following Varker et al. (2020), who recommended thresholds between 30-50% symptom reduction, we will define response as a 30% reduction in baseline symptom severity to ensure comparability across different measures while maintaining sensitivity. We will then conduct sensitivity analyses using the response rates as defined in the respective study and using a more conservative 50% symptom reduction threshold, aligned with recent meta-analytical approaches (Cuijpers et al., 2024).“

(Thank you again for bringing awareness to this important topic, that we also consider highly relevant for clinically relevant research; we even have studies in preparation that further explore these key issues.)

4. line 103: I think a more comprehensive discussion on augmentation strategies and how they have been used in the past is in order at this point. The mention of the 2 reviews seems to introductory, given this is the area of focus of this review. Expanding on the role of augmentation and maybe examples of augmentation from existing studies seems appropriate. Also discussing the moderators identified prior to setting the objectives describing the moderators seems indicated.

Reply: Thank you for suggesting a more comprehensive discussion on augmentation strategies. To address this and a similar comment from Reviewer 1 we have added more background on some examples and past uses for nonpharmacological augmentation strategies. We hope these revisions adequately address your concerns:

In contrast, nonpharmacological augmentation strategies could offer advantages through their more comprehensive therapeutic pathways: First, they could enhance different core TFP mechanisms, as demonstrated for example by exercise promoting neural plasticity (Powers et al., 2015)*, and breathing feedback supporting emotional arousal regulation during exposure* (Rosaura Polak et al., 2015)*. Second, they could address additional therapeutic targets not explicitly included in TFPs and hereby interact with the overall therapy process rather than specific mechanisms, such as sleep-directed interventions addressing common comorbid difficulties* (Galovski et al., 2016)*."*

In addition, thank you for bringing attention to the issue of moderator introduction before setting the objectives. Given the novelty of this research field and the expected limited number of studies, we approach moderator analyses as preliminary and hypothesis-generating rather than confirmatory. To maintain focus in the rationale, we have opted for a concise conceptual justification for examining moderators rather than a detailed theoretical discussion. To improve clarity, we have now briefly introduced the rationale for our moderator selection before outlining the study objective:

"However, these effects are unlikely to be uniform across all conditions, as the success of augmentation strategies may depend on factors such as type, dosage, integration within TFP, and patient characteristics. To gain insights for whom and under which conditions what specific augmentation could be most effective, we will explore potential moderators."

After careful reconsideration, we decided against using the previously prespecified moderator variables percentage of female participants and percentage of medicated participants for meta-regression due to concerns regarding ecological fallacy associated with study-level aggregates (Harrer et al., 2021; Thompson & Higgins, 2002).

The methods section is well-written.

I look forward to the results of this review.

Reply: We sincerely thank Reviewer 3 for their constructive feedback. Their suggestions have helped us improve both the theoretical foundation and methodological precision of our protocol through important clarifications and contextualization's throughout the manuscript.

References

Blevins, C. A., Weathers, F. W., Davis, M. T., Witte, T. K., & Domino, J. L. (2015). The Posttraumatic Stress Disorder Checklist for *DSM-5* (PCL-5): Development and Initial Psychometric Evaluation. *Journal of Traumatic Stress*, *28*(6), 489–498. https://doi.org/10.1002/jts.22059

Burback, L., Brémault-Phillips, S., Nijdam, M. J., McFarlane, A., & Vermetten, E. (2024). Treatment of Posttraumatic Stress Disorder: A State-of-the-art Review. *Current Neuropharmacology*, *22*(4), 557–635. https://doi.org/10.2174/1570159X21666230428091433

Cuijpers, P., Miguel, C., Ciharova, M., Harrer, M., Basic, D., Cristea, I. A., De Ponti, N., Driessen, E., Hamblen, J., Larsen, S. E., Matbouriahi, M., Papola, D., Pauley, D., Plessen, C. Y., Pfund, R. A., Setkowski, K., Schnurr, P. P., Van Ballegooijen, W., Wang, Y., … Karyotaki, E. (2024). Absolute and relative outcomes of psychotherapies for eight mental disorders: A systematic review and meta-analysis. *World Psychiatry*, *23*(2), 267–275. https://doi.org/10.1002/wps.21203

Deeks, J. J., Higgins, J. P., & Altman, D. G. (2023). Chapter 10: Analysing data and undertaking meta-analyses. In *Cochrane Handbook for Systematic Reviews of Interventions*.

Galovski, T. E., Harik, J. M., Blain, L. M., Elwood, L., Gloth, C., & Fletcher, T. D. (2016). Augmenting cognitive processing therapy to improve sleep impairment in PTSD: A randomized controlled trial. *Journal of Consulting and Clinical Psychology*, *84*(2), 167–177. https://doi.org/10.1037/ccp0000059

Hamblen, J. L., Norman, S. B., Sonis, J. H., Phelps, A. J., Bisson, J. I., Nunes, V. D., Megnin-Viggars, O., Forbes, D., Riggs, D. S., & Schnurr, P. P. (2019). A guide to guidelines for the treatment of posttraumatic stress disorder in adults: An update. *Psychotherapy*, *56*(3), 359–373. https://doi.org/10.1037/pst0000231

Harrer, M., Cuijpers, P., Furukawa, T. A., & Ebert, D. D. (2021). *Doing Meta-Analysis with R: A Hands-On Guide* (1st ed.). Chapman and Hall/CRC. https://doi.org/10.1201/9781003107347

Keyan, D., Garland, N., Choi-Christou, J., Tran, J., O'Donnell, M., & Bryant, R. A. (2024). A systematic review and meta-analysis of predictors of response to trauma-focused psychotherapy for posttraumatic stress disorder. *Psychological Bulletin*, *150*(7), 767–797. https://doi.org/10.1037/bul0000438

Lewis, C., Roberts, N. P., Gibson, S., & Bisson, J. I. (2020). Dropout from psychological therapies for post-traumatic stress disorder (PTSD) in adults: Systematic review and meta-analysis. *European Journal of Psychotraumatology*, *11*(1), 1709709. https://doi.org/10.1080/20008198.2019.1709709

Loerinc, A. G., Meuret, A. E., Twohig, M. P., Rosenfield, D., Bluett, E. J., & Craske, M. G. (2015). Response rates for CBT for anxiety disorders: Need for standardized criteria. *Clinical Psychology Review*, *42*, 72–82. https://doi.org/10.1016/j.cpr.2015.08.004

Martin, A., Naunton, M., Kosari, S., Peterson, G., Thomas, J., & Christenson, J. K. (2021). Treatment Guidelines for PTSD: A Systematic Review. *Journal of Clinical Medicine*, *10*(18), 4175. https://doi.org/10.3390/jcm10184175

Metcalf, O., Stone, C., Hinton, M., O'Donnell, M., Hopwood, M., McFarlane, A., Forbes, D., Kartal, D., Watson, L., Freijah, I., & Varker, T. (2020). Treatment augmentation for posttraumatic stress disorder: A systematic review. *Clinical Psychology: Science and Practice*, *27*(1). https://doi.org/10.1111/cpsp.12310

Nijdam, M. J., Vermetten, E., & McFarlane, A. C. (2023). Toward staging differentiation for posttraumatic stress disorder treatment. *Acta Psychiatrica Scandinavica*, *147*(1), 65–80. https://doi.org/10.1111/acps.13520

Powers, M. B., Medina, J. L., Burns, S., Kauffman, B. Y., Monfils, M., Asmundson, G. J. G., Diamond, A., McIntyre, C., & Smits, J. A. J. (2015). Exercise Augmentation of Exposure Therapy for PTSD: Rationale and Pilot Efficacy Data. *Cognitive Behaviour Therapy*, *44*(4), 314–327. https://doi.org/10.1080/16506073.2015.1012740

Rosaura Polak, A., Witteveen, A. B., Denys, D., & Olff, M. (2015). Breathing Biofeedback as an Adjunct to Exposure in Cognitive Behavioral Therapy Hastens the Reduction of PTSD Symptoms: A Pilot Study. *Applied Psychophysiology and Biofeedback*, *40*(1), 25–31. https://doi.org/10.1007/s10484-015-9268-y

Schnyder, U., Ehlers, A., Elbert, T., Foa, E. B., Gersons, B. P. R., Resick, P. A., Shapiro, F., & Cloitre, M. (2015). Psychotherapies for PTSD: What do they have in common? *European Journal of Psychotraumatology*, *6*(1), 28186. https://doi.org/10.3402/ejpt.v6.28186

Sterne, J. A. C., Savović, J., Page, M. J., Elbers, R. G., Blencowe, N. S., Boutron, I., Cates, C. J., Cheng, H.-Y., Corbett, M. S., Eldridge, S. M., Emberson, J. R., Hernán, M. A., Hopewell, S., Hróbjartsson, A., Junqueira, D. R., Jüni, P., Kirkham, J. J., Lasserson, T., Li, T., … Higgins, J. P. T. (2019). RoB 2: A revised tool for assessing risk of bias in randomised trials. *BMJ (Clinical Research Ed.)*, *366*, l4898. https://doi.org/10.1136/bmj.l4898

Thompson, S. G., & Higgins, J. P. T. (2002). How should meta-regression analyses be undertaken and interpreted? *Statistics in Medicine*, *21*(11), 1559–1573. https://doi.org/10.1002/sim.1187

Torbecke, J., Langhammer, T., Mewes, L., Lueken, U., & Fendel, J. C. (2024). Augmentation of cognitive-behavioural therapy for obsessive-compulsive and anxiety disorders: A protocol for a systematic review and meta-analysis. *BMJ Open*, *14*(10), e090431. https://doi.org/10.1136/bmjopen-2024-090431

Varker, T., Jones, K. A., Arjmand, H.-A., Hinton, M., Hiles, S. A., Freijah, I., Forbes, D., Kartal, D., Phelps, A., Bryant, R. A., McFarlane, A., Hopwood, M., & O'Donnell, M. (2021). Dropout from guideline-recommended psychological treatments for posttraumatic stress disorder: A systematic review and meta-analysis. *Journal of Affective Disorders Reports*, *4*, 100093. https://doi.org/10.1016/j.jadr.2021.100093

Varker, T., Kartal, D., Watson, L., Freijah, I., O'Donnell, M., Forbes, D., Phelps, A., Hopwood, M., McFarlane, A., Cooper, J., Wade, D., Bryant, R., & Hinton, M. (2020).

Defining response and nonresponse to posttraumatic stress disorder treatments: A systematic review. *Clinical Psychology: Science and Practice*, *27*(4). https://doi.org/10.1037/h0101781

Weathers, F. W., Bovin, M. J., Lee, D. J., Sloan, D. M., Schnurr, P. P., Kaloupek, D. G., Keane, T. M., & Marx, B. P. (2018). The Clinician-Administered PTSD Scale for DSM–5 (CAPS-5): Development and initial psychometric evaluation in military veterans. *Psychological Assessment*, *30*(3), 383–395. https://doi.org/10.1037/pas0000486

VERSION 2 - REVIEW

**Reviewer**	**3**
**Name**	**Rossouw, Jaco**
**Affiliation**	**Centre for CBT**
**Date**	**30-Mar-2025**
**COI**	

I picked up 2 typos, in dicated in the attached marked copy.

Thank you to the authors for the thorough changes made to the manuscript.

I have no further comments.

**** **The reviewer provided a marked copy with additional comments. Please contact the publisher for full details.** ****
